# Periprosthetic Joint Infection Following Acupuncture Treatment in a Patient With Total Hip Arthroplasty: A Rare but Severe Complication

**DOI:** 10.1155/cro/7852835

**Published:** 2026-04-29

**Authors:** Akira Yuasa, Hiroki Kobayashi, Keisuke Horiuchi

**Affiliations:** ^1^ Department of Orthopedic Surgery, National Defense Medical College, Tokorozawa, Saitama, Japan, ndmc.ac.jp

**Keywords:** acupuncture therapy, arthroplasty replacement hip, continuous local antibiotic perfusion, prosthesis-related infections

## Abstract

An 81‐year‐old woman underwent total hip arthroplasty for a femoral neck fracture. Five days after surgery, she fell and sustained a periprosthetic fracture, for which she subsequently underwent revision surgery. Her postoperative course was uneventful; however, 4 years after surgery, she noticed redness and warmth over her left hip several days after receiving acupuncture. Purulent discharge subsequently developed, prompting her to visit our hospital. Imaging studies revealed abscess formation and osteolysis around the left hip, leading to a diagnosis of periprosthetic infection. After conservative treatments failed, surgical debridement and continuous local antibiotic perfusion therapy were performed. Her condition improved, and she was discharged home, ambulatory with a cane. Although serum inflammatory markers remained mildly elevated, suggesting a low‐grade infection, there has been no recurrence of purulent discharge, and she has returned to her usual daily activities. Periprosthetic infection is a highly challenging complication, often resulting in the removal of implants and leading to a significantly diminished quality of life. Although acupuncture is generally regarded as safe, this case illustrates that deep infection can occur in patients with prosthetic joints. While a complete cure was not achieved in this case, the use of CLAP therapy contributed to clinical improvement and functional recovery, highlighting its potential role as an adjunctive treatment strategy. Given the potential risks of infection, physicians should inform patients with artificial joints about the potential infectious risks of acupuncture.

## 1. Introduction

Periprosthetic joint infection (PJI) is a serious complication that can significantly compromise patients′ quality of life and ability to perform daily activities. In patients who underwent total hip arthroplasty (THA), the reported incidence of PJI occurring within 2 years and beyond 2 years after surgery is 0.3% and 0.1%, respectively [1]. Known risk factors for PJI include obesity, coronary artery disease, and pulmonary hypertension [1]. Diagnostic criteria for PJI were established based on the statements by the 2013 International Consensus Meeting on Periprosthetic Joint Infection, which emphasize clinical findings such as sinus tract formation, microbiological evidence, and laboratory markers [2]. In general, PJI is highly challenging to manage and often necessitates the removal of implants, resulting in significantly diminished quality of life. Various treatment strategies have been proposed for this severe condition in the past. Among them, continuous local antibiotic perfusion (CLAP) therapy has recently emerged as a promising adjunctive method [3, 4]; however, its use in patients with THA has been rarely studied.

Acupuncture is a traditional Chinese medicine that involves the insertion of thin needles into specific points on the body. Although its effectiveness remains controversial, acupuncture is often used to treat various conditions and relieve pain. While the procedure is generally regarded as safe, there are reports of severe complications associated with acupuncture, including infective endocarditis, septic pulmonary embolism, and fatal pneumothorax, as well as deep infections in patients with joint replacements [5–8]. These reports suggest that acupuncture poses a risk of various complications, particularly infection. However, this risk may not be fully recognized and overlooked by patients and acupuncture practitioners.

Here, we present a case of PJI that developed 4 years after THA, most likely caused by acupuncture. Although the infection was not completely eradicated, the patient was successfully treated with a combination of surgical debridement and CLAP therapy without removing the prosthesis. Taken together, this report highlights the potential effectiveness of CLAP therapy as an adjunctive treatment for patients with hip PJI and raises awareness of this rare but severe complication of acupuncture.

## 2. Case Presentation

**Figure 1:**
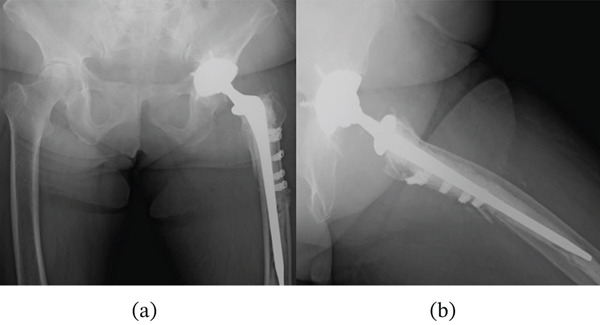
Postoperative radiographs after revision THA: (a) anteroposterior view and (b) lateral view.

An 81‐year‐old woman presented with redness and pain in the left proximal thigh. Four years earlier (Year X‐4), she underwent THA due to a femoral neck fracture. However, 5 days postoperatively, she fell in the hospital, resulting in a periprosthetic fracture. The fracture was classified as Vancouver Type B3, rendering stem retention unfeasible. Revision surgery was subsequently performed using a cemented long stem and cerclage cables (Figure 1). Postoperative rehabilitation was resumed, and her gait stabilized with cane assistance, allowing for discharge home. The following 4 years of postoperative follow‐up were uneventful.

In May of Year X, the patient underwent acupuncture for muscle stiffness in the left proximal thigh. According to her account, the procedure was performed under aseptic conditions. The treatment was repeated several times, and on multiple occasions, the needles were inserted until they appeared to reach the bone. The type of needles used and whether they were sterilized were unknown. Several days later, she noticed redness around the acupuncture insertion sites. The redness and warmth of the skin gradually worsened over time; however, she chose not to seek medical attention. By July of the same year, purulent discharge developed at the insertion site, and the patient experienced increasing difficulty walking, prompting her to visit a local clinic. Clinical and physical findings strongly suggested PJI. She was prescribed oral minocycline (200 mg/day) and referred to our institution for further evaluation.

**Figure 2:**
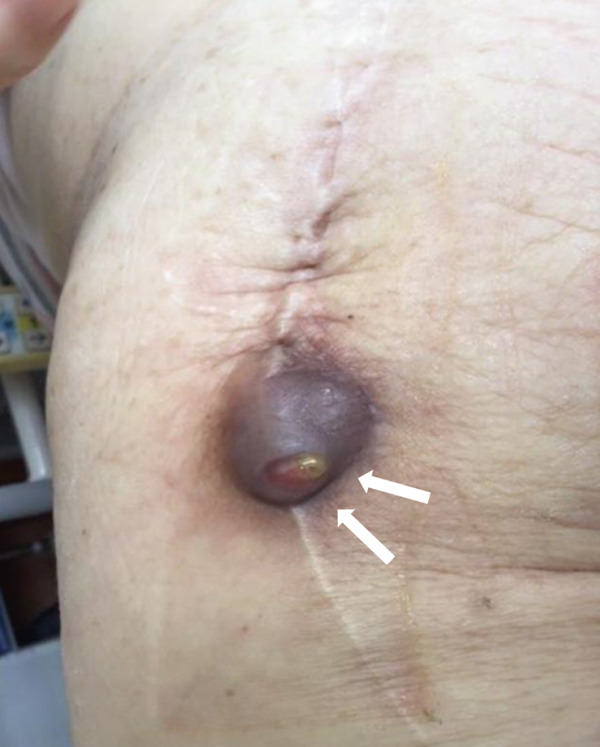
A macroscopic photograph showing the skin of the left thigh at the initial visit. Purulent discharge and mild erythema are noted at the needle insertion sites (arrow).

**Figure 3:**
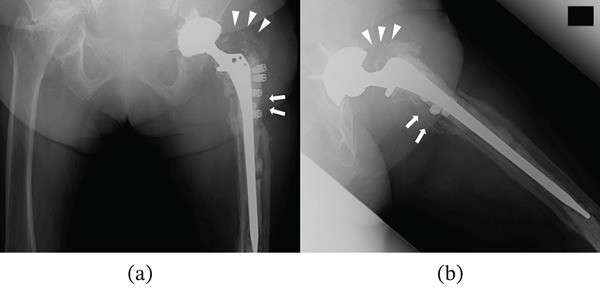
Radiographs at the initial visit: (a) anteroposterior view and (b) lateral view. Extensive osteolysis is seen in the proximal lateral region of the femur, where the infection has spread (arrowheads). The wires are loosened and have been dislocated from their original positions (arrow).

**Figure 4:**
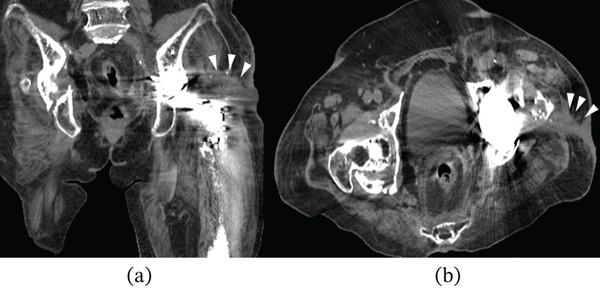
Preoperative computed tomography images showing the abscess formed around the proximal femur: (a) horizontal section and (b) axial section. Note the extensive osteolytic changes in the proximal lateral region of the femur (arrowheads).

Her past medical history included bilateral total knee arthroplasty for osteoarthritis, cervical cancer, and osteoporosis. She had no other comorbidities, such as diabetes, coronary artery disease, pulmonary hypertension, or obesity (body mass index, 26.1 kg/m^2^). She had not received any immunosuppressive therapy or medication that could potentially cause immunosuppression. Upon admission, the patient was afebrile; however, redness around the insertion sites and persistent purulent discharge were noted (Figure 2). Radiographic and CT imaging revealed an abscess in the proximal thigh and extensive osteolysis of the lateral aspect of the proximal femur (Figures 3 and 4). Serum inflammatory markers were elevated, including a white blood cell count of 7500/*μ*L, a neutrophil proportion of 65.2%, and a C‐reactive protein level of 1.39 mg/dL. Based on these findings, a diagnosis of PJI was made, and intravenous administration of cefazolin (1 g every 8 h) and minocycline (100 mg every 12 h) was initiated. Erythrocyte sedimentation rate (ESR) was not measured at presentation. Preoperative joint aspiration was not performed because continuous purulent discharge from a sinus tract communicating with the prosthesis was present, which fulfills a major diagnostic criterion for PJI according to the 2013 International Consensus Meeting definition. In addition, the patient had already received oral antibiotics prior to referral, making it impractical to secure an adequate antibiotic‐free interval before aspiration. Despite negative cultures from both wound discharge and blood samples, local infection control was not achieved, necessitating surgical intervention on Hospital Day 10.

Intraoperatively, abscesses were identified around the proximal femur and hip joint, accompanied by extensive osteolysis that exposed the stem and bone cement. A sinus tract was also identified and confirmed to communicate directly with the prosthesis. Although abscesses were also noted surrounding the stem, the patient strongly opposed stem removal prior to surgery; therefore, the stem was retained. To enhance infection control, CLAP was applied as an adjunctive treatment, with the catheter tip positioned intra‐articularly. During surgery, multiple tissue samples were obtained from the periprosthetic tissue and abscess cavities for aerobic and anaerobic cultures. All cultures remained negative after standard incubation periods. Histopathological examination was not performed. These negative culture results were considered to be influenced by prior antibiotic exposure and possible biofilm‐associated infection. CLAP therapy was initiated postoperatively using gentamicin (1200 mg every 24 h), and systemic antibiotic therapy was switched to daptomycin (250 mg every 24 h) and rifampin (450 mg/day). By Postoperative Day 10, improvement in inflammatory markers allowed for the removal of the CLAP drainage system. Oral antibiotic therapy was initiated on Postoperative Day 17, consisting of levofloxacin (500 mg/day), minocycline (200 mg/day), and rifampin (450 mg/day). CLAP therapy was initiated using gentamicin to achieve a high local antibiotic concentration targeting biofilm‐associated infection while minimizing systemic exposure. Renal function was closely monitored during treatment, and no deterioration in serum creatinine levels was observed. No clinical signs or symptoms suggestive of auditory or vestibular dysfunction were reported. Systemic antibiotics were selected empirically to cover common Gram‐positive organisms, including biofilm‐forming bacteria, and were adjusted based on clinical response rather than culture results. The dosages of the other antibiotics were determined based on the patient′s body weight and renal function.

On Postoperative Day 21, the patient regained the ability to walk with a cane and was discharged home. Although prolonged oral antibiotic therapy had been planned, the patient self‐discontinued treatment approximately 60 days later due to nausea. C‐reactive protein levels remained mildly elevated at approximately 1–2 mg/dL, suggesting a persistent low‐grade infection. However, the patient reports no pain or purulent discharge at the surgical site and resumed her usual daily activities.

## 3. Discussion

Complications associated with acupuncture are well documented, ranging from infective endocarditis and septic pulmonary embolism [5] to fatal cases of pneumothorax [6], underscoring the potential for life‐threatening outcomes in patients receiving this procedure. In the orthopedic field, reported complications include nontuberculous mycobacterial infections following elbow acupuncture [7] and implant infections in patients with artificial knee replacements after acupuncture [8]. Given that acupuncture involves inserting needles deep into the body, it could inadvertently cause a deep infection by introducing pathogens, especially in patients with artificial implants and joints. Reflecting these concerns, Japanese guidelines for knee osteoarthritis explicitly advise against the use of acupuncture. In the present case, acupuncture performed after THA resulted in the development of PJI, underscoring the significant risks of postoperative infection in patients with artificial joints. It is also worth noting that acupuncture is a form of alternative medicine for which robust scientific evidence supporting efficacy remains limited [9, 10].

A correct and timely diagnosis of PJI is often difficult since its clinical features resemble those of other conditions, including cellulitis, superficial abscess, and aseptic loosening. The 2013 International Consensus Meeting on Periprosthetic Joint Infection proposed global diagnostic criteria for identifying infections associated with joint replacement surgery [2]. According to these criteria, a diagnosis of postoperative infection can be confirmed if either (i) a sinus tract communicating with the implant is present or (ii) the same microorganism is detected in two separate specimens from the affected joint (e.g., joint fluid or tissue). In the present case, we were not able to detect microorganisms in any of the specimens; however, the presence of a sinus tract communicating with the implant enabled a diagnosis of PJI. While the exact reason for the repeated negative cultures remains unclear, it could be explained by prior antibiotic therapy administered at the referring clinic before presentation to our hospital or by the presence of anaerobic or fastidious organisms, both of which are frequently implicated in culture‐negative PJI [11]. In addition, the persistence of infection in our case may be explained by biofilm formation on the prosthetic surface and the potential emergence of antibiotic‐resistant organisms. Biofilms are known to protect bacteria from host immunity and antimicrobial agents, thereby allowing chronic infection to persist despite prolonged antibiotic therapy [12, 13]. Other causes of late PJI, including hematogenous spread from other infectious foci and spontaneous late‐onset infection unrelated to acupuncture, were considered. However, the close temporal relationship between acupuncture and symptom onset, together with the anatomical correspondence between the needle insertion sites and the location of infection, makes these alternatives less likely.

The 2‐month delay between symptom onset and diagnosis in our case underscores the challenges in identifying PJI in a timely manner. Had purulent discharge from the sinus tract not been observed, the case would not have met the major diagnostic criteria. Coupled with negative culture results, this would have rendered the diagnosis particularly difficult. In addition, it is often challenging to identify the exact cause of deep infection, and in our case, a definitive causal link between the acupuncture and the onset of PJI cannot be fully established. Nevertheless, the close temporal relationship between the acupuncture procedure and the onset of symptoms, along with the anatomical concordance between the needle insertion site and the location of infection, makes alternative explanations highly unlikely.

The management of chronic PJI remains a significant challenge in the field of orthopedics, prompting exploration into various treatment strategies. Among these, CLAP therapy has garnered attention due to its promising outcomes in recent studies [3, 4]. This approach involves the sustained administration of high‐concentration antibiotics directly to the site of infection, effectively targeting both bone and soft tissue infections. While primarily noted for its effectiveness in treating fracture‐related infections, CLAP has also shown encouraging results in managing periprosthetic infections. Reports highlight its successful application in infections following total talus replacement [14], as well as its role in retaining implants during acute and chronic infections after total knee arthroplasty [15]. Compared with the established two‐stage revision arthroplasty, which remains the gold standard for infection eradication but requires multiple surgeries [16, 17], CLAP offers a less invasive alternative that allows for implant retention and an earlier functional recovery, thereby helping to maintain patients′ quality of life.

Although complete eradication of the infection was not achieved in our case, the use of CLAP contributed substantially to a favorable clinical outcome. This suggests that CLAP may be particularly useful for elderly or high‐risk patients who are unwilling or unsuitable to undergo major revision surgery. Future studies should further evaluate the role of CLAP in the management of implant‐associated infections, ideally through prospective trials comparing its efficacy with established approaches such as two‐stage revision, and should also clarify optimal patient selection, antibiotic regimens, and treatment duration [18].

## 4. Conclusion

The present case highlights a rare but serious complication associated with acupuncture and the use of CLAP as a potentially effective adjunctive therapy for patients with PJI. Management of PJI can be challenging and often necessitates a significant burden on patients. Since this complication can be avoided with prior knowledge, physicians should inform patients with joint implants about the potential infectious risks of acupuncture, particularly when performed near the surgical site.

## Funding

No funding was received for this manuscript.

## Ethics Statement

Institutional Review Board approval was waived because this report describes a single retrospective case and does not include identifiable personal information.

## Consent

Written informed consent for publication of this case report and accompanying images was obtained from the patient.

## Conflicts of Interest

The authors declare no conflicts of interest.

## Data Availability

The data that support the findings of this study are available from the corresponding author upon reasonable request.
